# Strategies for Fermentation Medium Optimization: An In-Depth Review

**DOI:** 10.3389/fmicb.2016.02087

**Published:** 2017-01-06

**Authors:** Vineeta Singh, Shafiul Haque, Ram Niwas, Akansha Srivastava, Mukesh Pasupuleti, C. K. M. Tripathi

**Affiliations:** ^1^Microbiology Division, Council of Scientific and Industrial Research - Central Drug Research InstituteLucknow, India; ^2^Department of Biotechnology, Institute of Engineering and TechnologyLucknow, India; ^3^Department of Biosciences, Jamia Millia Islamia (A Central University)New Delhi, India; ^4^Research and Scientific Studies Unit, College of Nursing and Allied Health Sciences, Jazan UniversityJazan, Saudi Arabia; ^5^Fermentation Technology Division, Council of Scientific and Industrial Research - Central Drug Research InstituteLucknow, India; ^6^Department of Biotechnology, Shri Ramswaroop Memorial UniversityLucknow, India

**Keywords:** media optimization, OFAT, RSM, ANN, genetic algorithm

## Abstract

Optimization of production medium is required to maximize the metabolite yield. This can be achieved by using a wide range of techniques from classical “one-factor-at-a-time” to modern statistical and mathematical techniques, viz. artificial neural network (ANN), genetic algorithm (GA) etc. Every technique comes with its own advantages and disadvantages, and despite drawbacks some techniques are applied to obtain best results. Use of various optimization techniques in combination also provides the desirable results. In this article an attempt has been made to review the currently used media optimization techniques applied during fermentation process of metabolite production. Comparative analysis of the merits and demerits of various conventional as well as modern optimization techniques have been done and logical selection basis for the designing of fermentation medium has been given in the present review. Overall, this review will provide the rationale for the selection of suitable optimization technique for media designing employed during the fermentation process of metabolite production.

## Introduction

Fermentation technology is widely used for the production of various economically important compounds which have applications in the energy production, pharmaceutical, chemical and food industry. Although, fermentation processes are used from generations, the need for sustainable production of products, meet the market requirements in a cost effective manner has put forward a challenging demand. For any fermentation based product, the most important thing is the availability of fermented product equal to that of market demand. Various microorganisms have been reported to produce an array of primary and secondary metabolites, but in a very low quantity. In order to meet the market demand, several high yielding techniques have been discovered in the past, and successfully implemented in various processes, like production of primary or secondary metabolites, biotransformation, oil extraction etc. (Dubey et al., 2008, 2011; Singh et al., 2009; Rajeswari et al., 2014).

**Figure 1:**
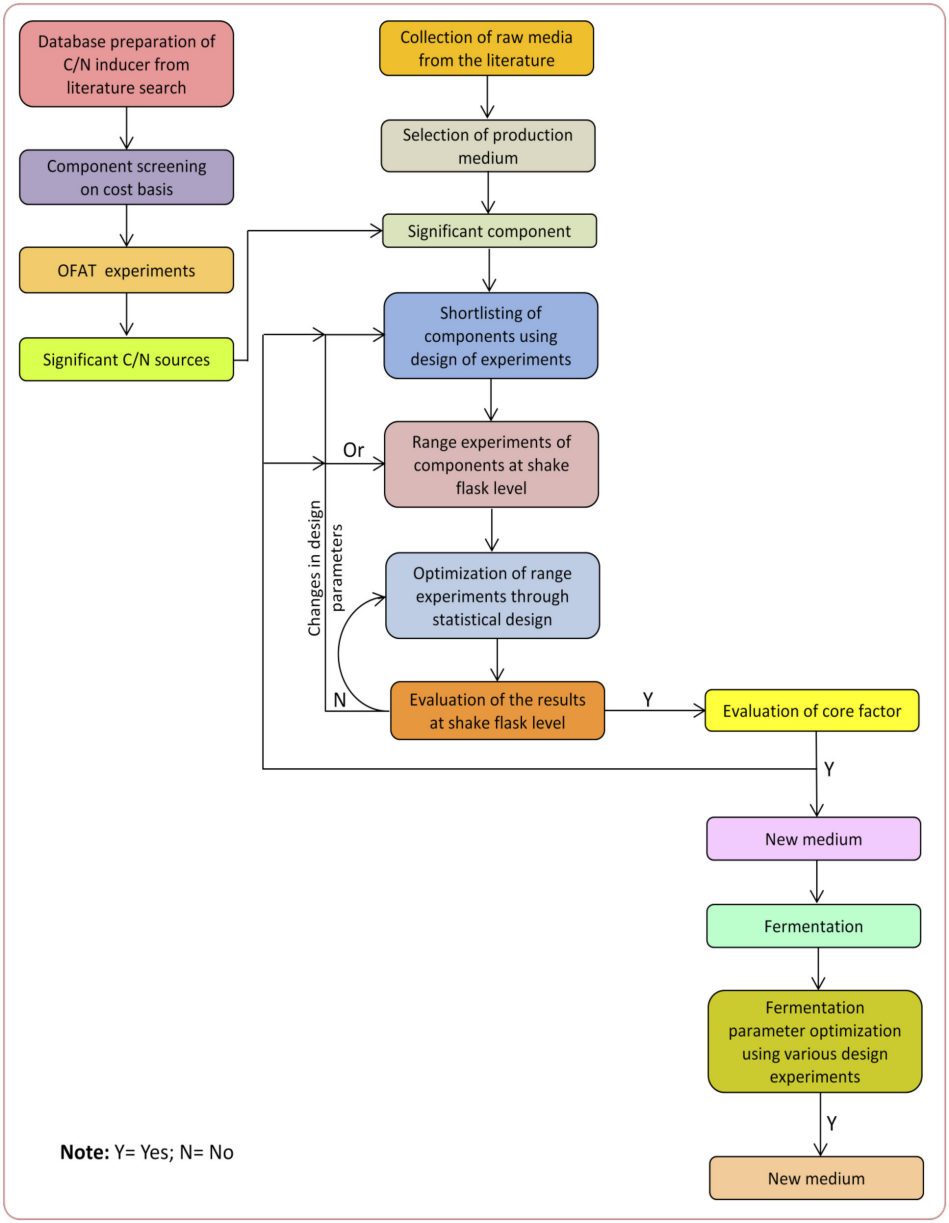
**Schematic diagram of a systematic approach of fermentation medium designing**.

Medium optimization is still one of the most critically investigated phenomenon that is carried out before any large scale metabolite production, and possess many challenges too. Before 1970s, media optimization was carried out by using classical methods, which were expensive, time consuming, involving plenty of experiments with compromised accuracy. Nevertheless, with the advent of modern mathematical/statistical techniques, media optimization has become more vibrant, effective, efficient, economical and robust in giving the results. For designing a production medium, the most suitable fermentation conditions (e.g., pH, temperature, agitation speed, etc.) and the appropriate medium components (e.g., carbon, nitrogen, etc.) must be identified and optimized accordingly. Further, by optimizing the above said parameters, maximum product concentration could be achieved (Gupte and Kulkarni, 2003; Franco-Lara et al., 2006; Wang et al., 2011). The schematic representation of a systematic approach of fermentation medium designing has been given in Figure 1.

An increase in productivity reduces the overall cost of the product, as well as the production cost; hence, it is one of the important topics for the research. Usually, enhanced productivity can be achieved either by strain improvement or by optimizing the process parameters. But, strain improvement and optimization are “Catch-22” situation. You cannot chose a lead strain until you have the best medium and you cannot propose a finest medium until you have the lead strain. Usually, the researchers around the world solve this predicament by sticking to one component at a time. However, both strategies cannot guarantee that one of the preferred strain if another medium is used. With this drawback and Catch-22 situation, various new methods have been suggested and investigated, where both the medium design and strain improvement can be carried out simultaneously.

In this review we have restricted our scope and discussed about the media formulation and media optimization techniques in terms of their utility, application and feasibility to maximize the metabolite yield produced by the fermentation process. In order to provide clarity and better understanding for the readers, initially we have discussed the roles of various (major) components of the fermentation media, followed by detailed description of statistical/mathematical optimization techniques. Also, the advantages and disadvantages associated with the above methods along with the future directions in the fermentation media design and optimization have been discussed in detail.

## Nutritional control of metabolite production

Fermented products that are used in our daily life are either primary or secondary metabolites produced during the trophophase and idiophase of the microbial growth, respectively. High productivity titer is the pre-requisite for the industrial production of any type of metabolite. The production of specific metabolites in high titer could be possible by maintaining proper control and regulation at different levels via transport and metabolism of extra-cellular nutrients, precursor formation and accumulation of intermediates (Rokem et al., 2007). Fermentation processes, where the precursor(s) of the specific products are not added in the medium, carbon and nitrogen sources present in the medium during their metabolism may initiate the biosynthesis of precursors that regulate the metabolism and influence the end product synthesis (Elibol, 2004). Given this in view, nutrients type and their concentrations in the medium play an important role in commencing the production of primary and secondary metabolites as limited supply of an essential nutrient can restrict the growth of microbial cells or product formation. Generally, carbon and nitrogen sources present in the medium can influence the metabolite production.

### Carbon source

Carbon is the most important medium component, as it is an energy source for the microorganisms and plays an important role in the growth as well as in the production of primary and secondary metabolite. The rate at which the carbon source is metabolized can often influence the formation of biomass and/or the production of primary or secondary metabolites. Marwick et al. (1999), while studying antibiotics production from marine bacteria noticed that the gradually assimilating carbon sources, like, galactose generally enhances the production of secondary metabolites (antibiotics). A classic example for this is, penicillin production, where glucose is found to have repression effect. Later, it was found that lactose is a slowly assimilating carbon source and helped in the production of secondary metabolites (i.e., penicillin). Hence, in order to overcome the carbon catabolite repression phenomenon, the production process was established using lactose fermentation. Describing the role of each carbon in different fermentation processes, will increase the length of this manuscript. Hence we compiled a list, wherein we summarized some interfering and non-interfering carbon sources (Table 1).

**Table 1:** **Examples of some interfering and non-interfering carbon sources**.

**Carbon**	**Source**	**Action**	**Metabolites**	**Producer**	**References**
Simple carbon	Glycerol	Interfering	Actinomycin D	*Streptomyces parvullus*	Foster and Katz, 1981
			Erythromycins	*Saccharopolyspora erythraea*	Sánchez et al., 2010
			Cephalosporin	*Cephalosporium acremonium*	Sanchez and Demain, 2002
		Non-interfering	Simocyclinones	*Streptomyces antibioticus*Tü 6040	Theobald et al., 2000
Monosaccharide	Glucose	Interfering	Actinomycin	*Streptomyces* sp.	Gallo and Katz, 1972
			Cephalosporin	*Cephalosporium acremonium*	Sanchez and Demain, 2002
			Erythromycins	*Saccharopolyspora erythraea*	Sánchez et al., 2010
			Penicillin	*Streptomyces chrysogenum*	Sanchez and Demain, 2002
			Streptomycin	*Streptomyces griseus*	Sanchez and Demain, 2002
		Non-interfering	Bacilysin	*Bacillus subtilis*	Ozcengiz et al., 1990
	Fructose	Interfering	Penicillin	*Penicillium chrysogenum*	Sanchez and Demain, 2002
		Non-interfering	Actinomycin	*Streptomyces antibioticus*	Rokem et al., 2007
			Gentamycin	*Micromonospora purpurea*	Sanchez and Demain, 2002
	Galactose	Interfering	Penicillin	*Penicillium chrysogenum*	Sanchez and Demain, 2002
		Non-interfering	Actinomycin	*Streptomyces antibioticus*	Rokem et al., 2007
			Cephalosporin	*Cephalosporium acremonium*	Sanchez and Demain, 2002
Disaccharide	Maltose	Interfering	Bacilysin	*Bacillus subtilis*	Ozcengiz et al., 1990
		Non-interfering	Gentamycin	*Micromonospora purpurea*	Sanchez and Demain, 2002
	Sucrose	Interfering	Erythromycins	*Streptomyces erythreus*	Rokem et al., 2007
			Penicillin	*Penicillium chrysogenum*	Sanchez and Demain, 2002
		Non-interfering	Cephalosporin	*Cephalosporium acremonium*	Sanchez and Demain, 2002
	Lactose	Interfering	^*^		
		Non-interfering	Erythromycins	*Streptomyce serythreus*	Rokem et al., 2007
			Penicillin	*Penicillium chrysogenum*	Rokem et al., 2007
	Mannose	Interfering	Erythromycin	*Streptomyce serythreus*	Sanchez and Demain, 2002
			Streptomycin	*Streptomyces griseus*	Sánchez et al., 2010
		Non-interfering	Kanamycin	*Streptomyces kanamyceticus*	Sanchez and Demain, 2002
Complex	Starch	Interfering	^*^		
		Non-interfering	Kanamycin	*Streptomyces kanamyceticus*	Rokem et al., 2007

* *Not reported*.

Fermentation processes, where raw materials/medium components cover the significant portion of the product cost, selection of these things become an important task for the production companies. In addition to the rate of assimilation of carbon sources, the nature of carbon source also affects the type and amount of the product. An example of this is ethanol or single-cell protein production, where the raw materials contribute ~60–77% of the production cost; and the selling price of the product is determined largely by the cost of the carbon source. Methanol could be a very popular inexpensive carbon source for single-cell protein production, but being toxic to the cells even at low concentrations and low flash points, it can never be used in fermentation as media. Hence, not only the cost even the dynamics of the carbon source must be considered whether it plays a role as a substrate in fermentation process or not.

### Nitrogen source

Like carbon, the selection of nitrogen source and its concentration in the media also play a crucial role in metabolite production. The microorganism can utilize both inorganic and/or organic sources of nitrogen. Use of specific amino acids can increase the productivity in some cases and conversely, unsuitable amino acids may inhibit the synthesis of secondary metabolites (Marwick et al., 1999). Singh et al. (2009) during the optimization of actinomycin V production by *Streptomyces triostinicus* found that biosynthesis of actinomycin V involves tryptophan pathway and addition of amino acid tryptophan to the medium enhances the production. On the contrary, the same amino acid showed inhibitory effect in the production of candicidin from *Streptomyces griseus* (Sanchez and Demain, 2002). Nevertheless, it is confirmed that nitrogen molecules have inhibitory effect on the metabolite production in some cases, whereas, some enhancer effects of nitrogen have also been reported (Table 2).

**Table 2:** **Examples of some interfering and non-interfering nitrogen sources**.

**Nitrogen**	**Source**	**Action**	**Metabolites**	**Producer**	**References**
Inorganic	${\text{NH}}_{4}^{+}$	Interfering	Spiramycin	*Streptomyces ambofaciens*	Lebrihi et al., 1992
			Cephalosporin	*Cephalosporium acremonium*	Sanchez and Demain, 2002
			Erythromycin	*Streptomyces erythreus*	Rokem et al., 2007
			Streptomycin	*Streptomyces griseus*	Sanchez and Demain, 2002
			Tetracycline	*Streptomyces* spp.	Rokem et al., 2007; Vastrad and Neelagund, 2011
		Non-interfering	^*^		
	Nitrate	Interfering	Aflatoxin	*Aspergillus parasiticus*	Sanchez and Demain, 2002
		Non-interfering	Rifamycin	*Amycolatoposis mediterranei*	Sanchez and Demain, 2002
Organic	Urea	Interfering	Alternariol	*Alternaría alternata*	
		Non-interfering	^*^		
Amino acids	L-alanine	Interfering	Actinomycin	*Streptomyces antibioticus*	Rokem et al., 2007
			Bacilysin	*Bacillus subtilis*	Ozcengiz et al., 1990
		Non-interfering	^*^		
	L-arginine	Interfering	^*^		
		Non interfering	Cephalosporin	*Cephalosporium acremonium*	Sanchez and Demain, 2002
			Gramicidin S	Bacillus brevis	Poirier and Demain, 1981
	d,l-Aspartate	Interfering	Actinomycin D	*Streptomyces parvullus*	Foster and Katz, 1981
		Non-interfering	Streptothricin	*Streptomyces rochei*	Sanchez and Demain, 2002
	Leucine	Interfering	Monascus pigment	*Monascus* spp.	Lin and Demain, 1994
		Non-interfering	Chloramphenicol	*Streptomyces venezuelae*,	Rokem et al., 2007
	L-isoleucine	Interfering	Actinomycin D	*Streptomyces parvullus*	Foster and Katz, 1981
		Non-interfering	Spiramycin	*Streptomyces ambofaciens*	Lebrihi et al., 1992
	DL- phalanine	Interfering	Actinomycin	*Streptomyces antibioticus*	Rokem et al., 2007
		Non-interfering	Chloramphenicol	*Streptomyces venezuelae*,	Rokem et al., 2007
	L-proline	Interfering	Actinomycin D	*Streptomyces parvullus*	Foster and Katz, 1981
		Non-interfering	Streptomycin	*Streptomyces griseus*	Sanchez and Demain, 2002
	Tryptophan	Interfering	Candicidin	*Streptomyces griseus*	Sanchez and Demain, 2002
		Non-interfering	Actinomycin	*Streptomyces parvullus*	Foster and Katz, 1981

* *Not reported*.

### Phosphate

Phosphate is another basic component which is required for the production of phospholipids present in the microbial cell membranes, and for the production of nucleic acids. The amount of phosphate which must be added in the fermentation medium depends upon the composition of the broth and the need of the organism, as well as according to the nature of the desired product. For instance, some cultures will not produce secondary metabolites in the presence of phosphate, e.g., phosphatase, phytases etc. Sanchez and Demain (2002) reported that various secondary metabolites' production such as, actinorhodin, cephalosporin, clavulanic acid, streptomycin, tetracycline, vancomycin etc. is highly influenced by inorganic phosphate concentration present in the production medium. In most cases, lower concentration of phosphate is required for the initiation of the metabolite (antibiotic) production and beyond a certain concentration it suppresses the secondary metabolism and ultimately inhibits the production of primary or secondary metabolite. High phosphate concentration was reported to inhibit the production of teicoplanin, a glycopeptide antibiotic (Rokem et al., 2007).

From the above description it is clear that changes in carbon or nitrogen sources of the production medium or variation from their optimum required concentration, may affect the nature of the end product or its productivity. Therefore, the production medium with all the required components in appropriate concentration is required for the production of desired metabolite at large scale. In order to standardize the production medium, the concept of medium optimization has emerged.

## Need of medium optimization

Medium optimization studies are usually carried out in the chemical, food, and pharmaceutical industries, with respect to increase the yield and activity of the desired product. Currently, there is a very little knowledge available about the role of factors, their levels in controlling the metabolite (e.g., antibiotics, acids) production by different strains. In order to enhance the productivity of the metabolites (for e.g., antibiotics etc.), researchers investigated the nutritional requirements for the production of secondary metabolites and found that the nutritional requirements were varying from strain to strain (Shih et al., 2002; Singh et al., 2012). The quantity and quality of nutrients available and the ability to assimilate successfully are the major determinants of microbial nature and its metabolic activity. Hence, during the medium optimization it must be considered that a minimal growth requirement of the microorganism must be fulfilled for obtaining maximum production of metabolite(s). As the fermentation process progresses into lower-value, higher-volume chemicals, it becomes necessary to maximize the efficiency and minimize the production cost and waste by-products to compete effectively against the traditional methods.

## Media optimization strategies

**Figure 2:**
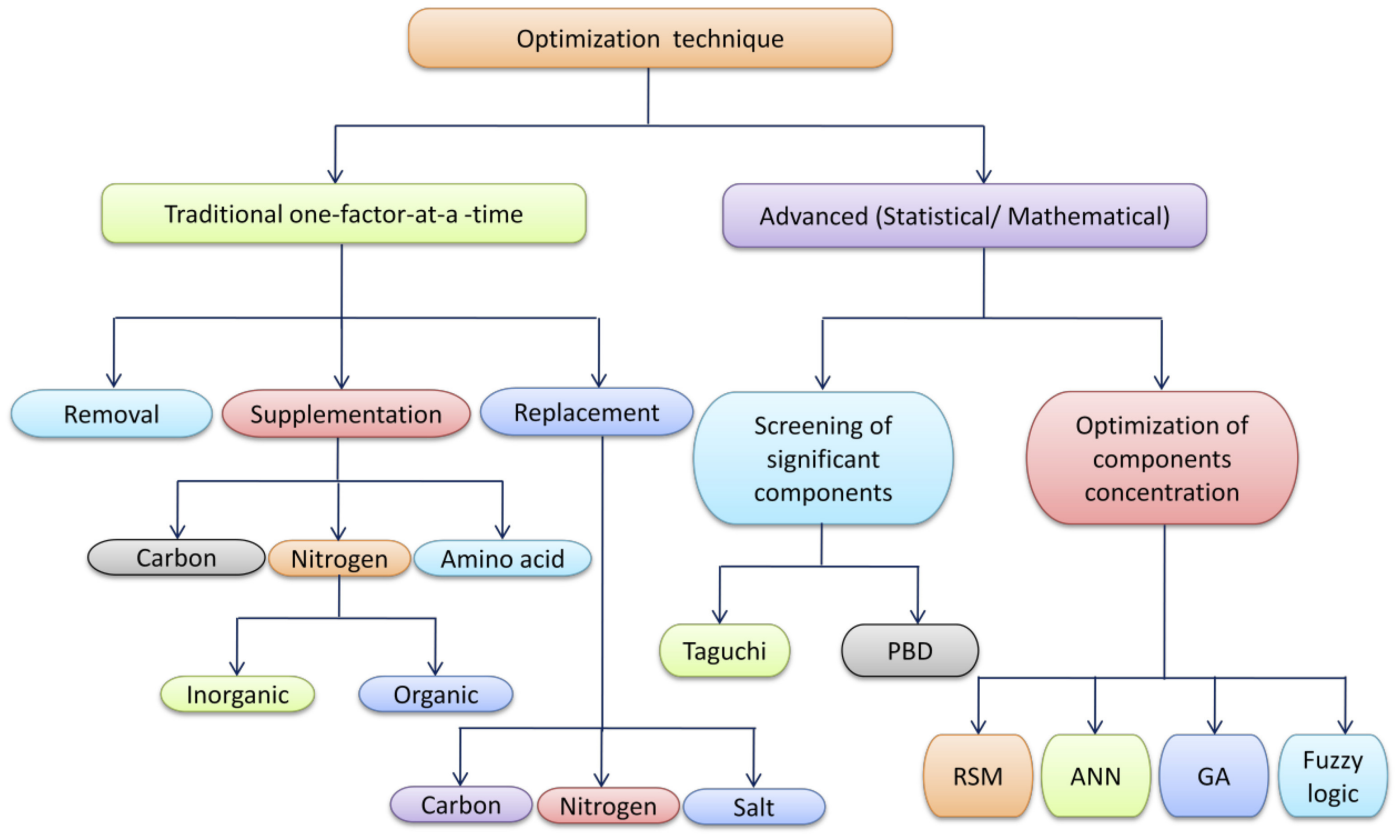
**Schematic representation of various techniques used in optimization studies**.

During the medium designing and optimization, there are various strategies available which are frequently used to improve the efficiency of the production medium. Figure 2 is a schematic representation of various techniques used in the medium optimization.

### Classical medium optimization methods

#### One-factor-at-a-time (OFAT)

In the classical medium optimization technique, one-factor-at-a-time (OFAT) experiments, only one factor or variable is varied at a time while keeping other variables constant. The concentrations of the selected medium components were then changed over a desired range. Because of its ease and convenience, the OFAT has been the most preferred choice among the researchers for designing the medium composition and used in the initial stages in diverse fields (Gonzalez et al., 1995). This methodology is still in use even today, during the initial stages of medium formulation for the production of new metabolite or known compound from new source. Based upon the approach applied, OFAT is further sub-grouped into:

##### Removal experiments

In this type of experiment, all the medium components are removed from the production medium one-by-one, and after proper incubation period, their effects on the production of secondary metabolite or the product of interest is observed in terms of suitable parameters. Our research group has previously reported that during the production of antifungal compound from *Streptomyces capoamus*, removal of soybean meal or glycerol or NaCl from the fermentation medium decreased the yield by 20–40% (Singh et al., 2008).

##### Supplementation experiments

Supplementation experiments are generally performed to evaluate the effects of various carbon and nitrogen supplements on metabolite production. During the study of antifungal production from *Streptomyces violaceusniger*, 70–90% enhancement in the yield was observed by supplementing xylose, sorbitol and hydroxyl proline in the production medium (Tripathi et al., 2004). Similarly, glycerol and peptone was found as a most suitable carbon and nitrogen sources for the production of antifungal and antibacterial metabolites from *Streptomyces rimosus* under submerged fermentation condition (Singh and Rai, 2012).

##### Replacement experiments

For medium formulation, carbon/nitrogen sources showing enhancement effect on the desired metabolite production in supplementation experiments are generally tried to be used as a whole carbon/nitrogen source.

##### Physical parameters

In addition to chemical and biological variables, several researchers used OFAT experiments to standardize the physical parameters such as pH, temperature, agitation and aeration requirements of the fermentation process (Niwas et al., 2013).

Like any other technique, OFAT method of medium optimization has its own advantages and disadvantages. The major advantage of OFAT is its simplicity by which a series of experiments can be carried out and results can be analyzed by using simple graphs without the aid of high end statistical analysis/programs. The major drawback of OFAT is the difficulty in estimating the “interactions” from the experiments as it is a hit-and-miss scattershot sequence of the experiments (Gupte and Kulkarni, 2003). Vaidya et al. (2003) described the time consumed and cost involved in the analysis of large number of variables as the major disadvantages of OFAT techniques. In this methodology, sometimes the optimum point may be missed completely, thus it requires a large number of experiments to determine the optimum level, which becomes laborious, time consuming, and uneconomical most of the time (Gupte and Kulkarni, 2003). Nevertheless, OFAT technique can be a best screening tool when nothing about the media is known because of its ease and convenience.

#### Design of experiments

The use of statistical method, i.e., design of experiments (DOE) for the media optimization in fermentation process can overcome the limitations of classical OFAT method and can be a powerful tool for the optimization of metabolite production. Fisher (1992) proposed a basic theory of experimental design which shows that changing more than one component in the medium at a time can be more efficient over changing only one-factor-at-a-time (Fisher, 1992).

DOE is a series of experiments which are strategically planned and executed to obtain a larger amount of information about the effect of more than one parameter at a time on the output, i.e., product yield. Most DOE procedures allow the preliminary screening of 2–10 medium factors in a limited number of experiments. In this method, several medium factors or components are compared simultaneously and the effects are observed and ranked based on the results. Once the response variables are determined and ranked, statistical performance parameters are generated from the subsequent analysis. Due to the requirement of higher number of experiments, OFAT is laborious, time consuming process, and extremely tedious for a large number of variables, whereas DOE requires fewer experiments, lesser time, and lesser material to obtain the same amount of information (Adinarayana and Ellaiah, 2002; Keskin Gündogdu et al., 2016). The interaction between the factors can be estimated systematically in DOE (Haaland, 1989). After getting the basic idea about the fermentation production process from the literature or from the classical experiments, designing of the experiments are more effective to determine the impact of two or more factors on a response than OFAT.

### Statistical medium optimization

With the advancement of statistical techniques, medium optimization has found new dimensions, as these techniques improve the efficiency of the process, reduces the time required in the process and labor cost etc., thus contributing toward the overall economics of the process. Being, biological in nature, the microbial processes contain relatively large amount of natural variations. The networks associated with the microbial reactions are complex, and several factors affect different parts of the networks. Rational experimental design and statistical evaluation of the results increase the knowledge about the reliability of the information obtained during the experiments. By using experimental design, the amount of experiments required to obtain a for reliable process optimization can be reduced (Elibol, 2004).

Many studies claim substantial improvements over media obtained using OFAT techniques by using DOE methods. For example, during the study of rate of methane and carbon dioxide gas production from *Methanosarcina barkeri* bacterium growing on methanol, medium optimized through experimental design was found to give 1.3 times more gas production as compared to the OFAT optimized medium (Silveira et al., 1991). Given this, it is widely accepted that in order to have an improved media by employing the experimental design approach; we require both a design as well as the optimization technique. The DOE defines the medium variants to be tested such as, number of replicates and the arrangement of the tests in a harmonized pattern etc. Based upon the obtained experimental data, optimization technique is used to predict a mathematical model and improve the medium composition.

#### Experimental design

Experimental design is a study plan to get defined goals or objectives. Modern statistical techniques provide us powerful tools for the evaluation of the components or variables effects based on the experimental results. Hence, the experiments must be planned properly with the sufficient sample size to obtain adequate data which is essential to answer the objective as efficiently as possible. Such types of techniques are commonly called as DOE. In a full factorial design, all the combinations of the factors, e.g., pH, strain, medium components, temperature etc. are tested. In contrast, in a partial factorial analysis, only few well reported combinations are picked-up and tested. Usually, partial factorial analysis is done, when the full factorial design is not possible and some or little knowledge about the interactions of the medium components for a particular strain is available.

##### Plakett burman design

All the components present in the medium do not contribute in the metabolite production. Hence, it is utmost important that the non-contributing factors, should be eliminated from the study as early as possible. In 1946, R.L. Plackett and J.P. Burman published their work entitled “*The design of optimal multifactorial experiments*” as a solution to determine the major effects with higher precision in any process. Plakett Burman Design (PBD), is a two-level design, which is very useful for economically detecting the main effects and assuming all the other interactions are negligible when comparing the some important major effects, i.e., when there are no interactions, the observed effect of a factor can be superior or under estimated by other factors (Vaidya et al., 2003). An example of PBD has been given in Table 3. PDB is used to screen “*n*” number of experimental variables in just “*n*+1” number of experiments (Reddy et al., 1999; Ghanem et al., 2000). In this design, there are two types of variables, i.e., “*real variables*” whose concentration changes during the experiments, and “*dummy variables*,” whose concentration remains constant during the experiments and used to estimate the error. Each variable is represented in two levels, i.e., high (H) and low (L). Each horizontal row represents a trial and each vertical column represents the either of two levels (high or low) of each independent and dummy variables in all the trials. Usually, the classical experiments help in the selection of independent and dummy variables. The effect of each variable is determined by the following equation:

$$\begin{array}{l} {\text{E}}_{\text{x}1}\;=2\left(\sum {\text{Y}}_{\text{x}1\text{H}}\;-\;\sum {\text{Y}}_{\text{x}1\text{L}}\right)/\text{N}; \end{array}$$

Where, E_(*X*1)_ = Effect of variable; Y_X1-*H*_ = yield from the trials having high concentration of variable; Y_*X*1−*L*_ = yield from the trials having low concentration of variable and N = total number of trials.

**Table 3:** **Plackett-Burman design for eleven variables**.

**Runs**	**Variables and levels**										
	***X*1**	***X*2**	***X*3**	***X*4**	***X*5**	***X*6**	***D*1**	***D2***	***D3***	***D4***	***D5***
1	L	H	L	L	L	H	H	H	L	H	H
2	H	L	L	L	H	H	H	L	H	H	L
3	L	L	L	H	H	H	L	H	H	L	H
4	L	L	H	H	H	L	H	H	L	H	L
5	L	H	H	H	L	H	H	L	H	L	L
6	H	H	H	L	H	H	L	H	L	L	L
7	H	H	L	H	H	L	H	L	L	L	H
8	H	L	H	H	L	H	L	L	L	H	H
9	L	H	H	L	H	L	L	L	H	H	H
10	H	H	L	H	L	L	L	H	H	H	L
11	H	L	H	L	L	L	H	H	H	L	H
12	L	L	L	L	L	L	L	L	L	L	L

*H, high conc. of the components; L, low conc. of the components; D, dummy variable*.

Experimental error is estimated by calculating the variance among the dummy variables as follows: V_eff_ = ∑(${\text{E}}_{\text{d}}^{2}$)/n; where V_eff_ = variance of the concentration effect, E_d_ = effect for dummy variable and n = number of dummy variables. The standard error (SE) of the concentration effect is the square root of the variance (√ V_eff_). The significance level of the effect of each variable is determined by student's *t*-test: t_x1_ = E_x1_/SE. The variables with confidence levels greater than 90–95% will be considered to influence the metabolite production significantly.

PBD is an authentic method to evaluate the relative importance of various variables or medium components for specific output, for e.g., antibiotic or other cellular metabolite production (Ghanem et al., 2000; Vaidya et al., 2003; Singh and Tripathi, 2008; Rajeswari et al., 2014). Use of PBD decreases the total number of experiments, tremendously (Adinarayana and Ellaiah, 2002), as the interaction effects of the variables not consider and only those variables that actually affect the production of desired metabolite are screened. For gamma interferon production using PBD, 20 medium components were examined in only 24 runs, and 45% higher production was observed (Castro et al., 1992). Likewise, during the initial studies of medium optimization for antibacterial metabolite production from *Streptomyces* sp, we have used PBD to identify the most effective components in the media and reported soybean meal, calcium carbonate, and potassium phosphate can significantly increase the antibiotic production (Banga et al., 2008).

Even though PBD is a good method to identify the important components, but there are some drawbacks associated with its efficiency. PBD should be used only when the factors have no interactions, or have only additive effects on the output, otherwise the results of the factor analyzed will be enhanced or masked by other factors as it fails to interpret if the effect of one factor depends on another factor. Nevertheless, in the DOE, PBD is a starting point and one should use it to determine the follow-up experimentation list. Given this, PBD is usually called “screening designs” because they help you to screen out non-contributing factors, i.e., for higher yield, from that of contributing factors.

##### Taguchi design

In order to overcome the problems associated with the PBD method, Dr. Genichi Taguchi developed a method which is based on “ORTHOGONAL ARRAY.” This method tells us how different parameters affect the yield in a small number of experiments instead of testing all the possible combinations, like, the factorial design. Taguchi technique offers three-stages of off-line quality control features, like system strategy, parameter designing and tolerance design phase (Pignatiello, 1988). The system strategy helps in finding the experimental levels of design features while parameter designing shows the factor level and provides the paramount effects of the process, whereas the tolerance design phase improves the elemental tolerance that considerably effect the product formation (Muhammad et al., 2014). This design helps in determining the factors affecting the product significantly with a minimum number of experiments, thus saving time and resources. Analysis of variance (ANOVA) on the collected data from the Taguchi DOE can be used to select the new parameter values to optimize the performance characteristic. During the execution of the experiment, at first the total degree of freedom is selected [overall mean always uses 1 degree of freedom (DOF); for each factor DOF = n − 1, where n = number of levels; for any two factor interaction DOF = (n_a_ − 1) (n_b_ − 1)] followed by the selection of standard orthogonal array (generally, the number of runs in orthogonal design is ≥ to the DOF). At the end of the experiment, the factors are assigned to appropriate columns. Unlike PBD, it analyses the main effect and two factor interactions. However, higher order interactions are assumed as negligible. Noise, i.e., uncontrolled variables of experiments is taken as focal point for the analysis. Uncontrolled variables (noises) generally cause the loss of the quality. This effect of noise can be removed by employing the Taguchi methodology (Aggarwal and Singh, 2005).

The Taguchi method becomes very helpful in measuring the quality by the deviation of a functional characteristic from its target value. The Taguchi approach is a fully developed method having advantage of saving experimental time, product cost and improving the quality as well which is a basic requirement for the optimization of any fermentation process (Chanin et al., 1990). Recently, Muhammad et al. (2014) applied Taguchi's statistical approach in the first step to optimize the production of novel thermostable polypeptide antibacterial compound from *Geobacillus pallidus* under different production conditions such as incubation period, temperature, pH, aeration rate, nitrogen, and carbon concentrations.

##### Central composite design

As PBD considers only main effects and ignores the interactions among the factors, therefore, a new design is required. Central composite design (CCD) was first described by Box and Wilson (1951). Nowadays it is widely used in response surface methodology (RSM) for building a second order (quadratic) model for the response variable without using a complete three-level factorial experiment. The design consists of three distinct sets of experimental runs (Table 5): *factorial design* in which the factors studied, each having two levels (+1 and −1); *center points*, where experimental runs having the median values of each factor used in the factorial design. This point is often replicated in order to improve the precision of the experiment; *star points*, experimental runs identical to the center points except for one factor, which will take on values both below and above the median of the two factorial levels. The numbers of star points are double the number of factors used in the design. On the basis of set of experiments and the level of factors, CCD are of three types: Circumcentered CCD (CCC), Inscribed CCD (CCI) and Face centered CCD (CCF) (Table 4).

**Table 4:** **Structural comparisons of CCD (CCC (CCI), CCF) and BBD for three factors**.

**CCC (CCI)**				**CCF**				**Box-Behnken**			
**Rep**	***X*1**	***X*2**	***X*3**	**Rep**	***X*1**	***X*2**	***X*3**	**Rep**	***X*1**	***X*2**	***X*3**
1	−1	−1	−1	1	−1	−1	−1	1	−1	−1	0
1	+1	−1	−1	1	+1	−1	−1	1	+1	−1	0
1	−1	+1	−1	1	−1	+1	−1	1	−1	+1	0
1	+1	+1	−1	1	+1	+1	−1	1	+1	+1	0
1	−1	−1	+1	1	−1	−1	+1	1	−1	0	−1
1	+1	−1	+1	1	+1	−1	+1	1	+1	0	−1
1	−1	+1	+1	1	−1	+1	+1	1	−1	0	+1
1	+1	+1	+1	1	+1	+1	+1	1	+1	0	+1
1	−1.682	0	0	1	−1	0	0	1	0	−1	−1
1	1.682	0	0	1	+1	0	0	1	0	+1	−1
1	0	−1.682	0	1	0	−1	0	1	0	−1	+1
1	0	1.682	0	1	0	+1	0	1	0	+1	+1
1	0	0	−1.682	1	0	0	−1	3	0	0	0
1	0	0	1.682	1	0	0	+1				
6	0	0	0	6	0	0	0				
Total Runs = 20				Total Runs = 20				Total Runs = 15			

*CCC, Circumcentered; CCI, Inscribed; CCF, Face centered; BBD, Box-Behnken*.

**Table 5:** **A summary of designs and optimization techniques used for the improvement of production media in some of the published studies**.

**Design**	**Technique**	**Fold increase**	**Metabolite**	**Producer**	**References**
PBD	^*^	1.45	Gamma interferon		Castro et al., 1992
CCD	RSM	8.0	Clortetracyclin, tetracycline	*Streptomyces aureofaciens*	Teruel et al., 1997
PBD	^*^	^*^	β-amylase, pullulanase	*Clostridium thermosulfurogenes*	Reddy et al., 1999
PBD	^*^	^*^	Xylanase	*Aspergillus terreus*	Ghanem et al., 2000
PBD, CCD	RSM	1.82	Compactin	*Penicillium citrinum*	Chakravarti and Sahai, 2002
CCD	RSM	3.7	Poly (γ-glutamic acid)	Bacillus *licheniformis*	Shih et al., 2002
full FD	RSM	^*^	Antifungal antibiotic	*Streptomyces chattanoogensis*	Gupte and Kulkarni, 2003
PBD, BBD	RSM	1.4	Chitinase	*Alealigenes xylosoxydans*	Vaidya et al., 2003
^*^	ANN, GA	1.15	Xylitol	*Candida mogii*	Baishan et al., 2003
Full FD	RSM	1.30	Antifungal antibiotic	*Thermomonospora* sp.	Gupte and Kulkarni, 2003
CCD	RSM	1.35	Actinorhodin	*Streptomyces coelicolor*	Elibol, 2004
CCD	RSM	^*^	Polyaccharide	*Pleurotus citrinopileatus*	Wang et al., 2005
	OFAT	1.82	Polyketide antibiotic	*Streptomyce*s*psammoticus*	Sujatha et al., 2005
CCD	OFAT, RSM	1.53, 1.32	Eucalyptene A, xyloketal A	*Xylaria* sp. 2508	Xiaobo et al., 2006
PBD, CCD	ANN, GA	1.25	Exopolysaccharide	*Lactobacillus plantarum*	Desai et al., 2006
PBD, CCD	RSM	10	Candicidin derivatives	*Streptomyces* sp.	Mao et al., 2007
Frac FD	RSM	2.80	Avilamycin	*Streptomyces viridochromogenes*	Zhu et al., 2007
PBD, Full FD	RSM	^*^	Pyruvic acid	*Torulopsis glabrata*	Zhang and Gao, 2007
PBD, CCD	RSM	8.00	Olivanic acid.	*Streptomyces olivaceus*	Singh and Tripathi, 2008
PBD, CCD	RSM	3.56	Actinomycin D	*Streptomyces sindenensis*	Praveen et al., 2008
PBD, CCD	RSM	2.37	Heparinase	*Aspergillus flavus*	Banga and Tripathi, 2009
CCD	ANN, GA	4.00	ActinomycinV	*Streptomyces triostinicus*	Singh et al., 2009
BBD	ANN, GA	8.30	Nisin	*Lactobacillus lactis*	Guo et al., 2010
CCD	RSM	10.0	Oxytetracycline	*Streptomyces rimosus*	Singh et al., 2012
CCD	ANN, GA	4.00	Actinomycin D	*Streptomyces sindenensis*	Khan et al., 2011
CCD	RSM	1.37	Antibiotic	*Xenorhabdus* bovienii	Wang et al., 2011
PBD, BBD	RSM	2.61	Milbemycin	*Streptomyces bingchenggensis*,	Baoxin et al., 2011
CCD	ANN, NMDS	1.12	Actinomycin D	*Streptomyces sindenensis*	Tripathi et al., 2012
PBD, BBD	RSM	1.78	Antibiotic	*Streptomyces* sp.	Rajeswari et al., 2014
CCD	RSM	1.44	Jiean-peptide	*Bacillus subtilis*	Zhong et al., 2014

BBD, Box-Behnken design; CCD, Central composite design; Frac FD, Fractional factorial design; Full FD, Full factorial design; RSM, Response surface methodology; ANN, Artificial neural network; GA, Genetic algorithm; NM, Nelder-Mead Simplex; NA, Neighborhood analysis; DT, Decision Tree technique;

* *Not reported*.

##### Box Behnken design

The Box-Behnken design is an alternate to CCD, it is independent of quadratic design, which does not contain an embedded factorial or fractional factorial design (Ferreira et al., 2007).

In this design, the treatment combinations are at the mid-points of the edges of the process space and at the center. These designs are rotatable (or near rotatable) and require 3 levels of each factor. The designs have limited capability for orthogonal blocking compared to the central composite designs.

Some of the frequently used fermentation media optimization design techniques in the last 25 years have been summarized in Table 5.

#### Optimization techniques

##### Response surface methodology (RSM)

During the development of pharmaceutical formulations various production mediums and process variables related to the productivity, safety and usefulness should be optimized. Real relationship between the medium parameters and productivity is very difficult to understand thus creates hurdles in optimizing the pharmaceutical formulation. Box and Wilson (1951) developed a method, RSM, which uses factorial designs to optimize the production processes of the desired metabolites. RSM is a sturdy, robust and efficient mathematical approach which includes statistical experimental designs and multiple regression analysis, for seeking the best formulation under a set of constrained equations. RSM has often been applied to optimize the formulation variables and optimization of fermentation process (Houck et al., 1995; Franco-Lara et al., 2006). Vaidya et al. (2003) used RSM for chitinase production from *Alealigenes xylosoxydans* and found 1.4-folds production enhancement. Shih et al. (2002) reported 3.7-folds increased production of poly (γ-glutamic acid) from *Bacillus licheniformis* by optimizing nutrient concentration using RSM. RSM was applied to optimized water-soluble polysaccharide production from *Pleurotus citrinopileatus* in submerged culture (Wang et al., 2005). In the field of antibiotics production, the use of this methodology was reported for chlortetracycline and tetracycline production with K-carrageenan immobilized *Streptomyces aureofaciens* with 8-folds increase in the antibiotic yield (Teruel et al., 1997); Likewise, Gouveia and his co researcher during the study of clavulanic acid production by *Streptomyces clavuligerus* reported nearly 2.6-folds enhancement in the yield, when RSM technique was employed for the medium optimization (Gouveia et al., 2001).

RSM employs several phases of optimization (Gupte and Kulkarni, 2003) and it can be performed in three basic steps, i.e., experiments designed for the screening of the factors followed by the path of steepest ascent/descent and finally quadratic regression model is fitted and optimized using canonical regression analysis method. One of the important inputs of RSM is representation of the yield, as a surface plot. It can provide multiple responses at the same time by considering the interactions between the variables, which is utmost necessary for designing and process optimization (Zhang and Gao, 2007). Since, the theoretical relationships between the independent and dependent variables are not clear, multiple regression analysis can be applied to predict the dependent variables on the basis of a second-order equation.

$$\begin{array}{l} Y\left(X\right)\;=\;a_{0}\;+\;\sum_{i=0}^{N}a_{i}X_{i}+\sum_{i<j}^{N}a_{ij}X_{i}X_{j}+\;\sum_{i=0}^{N}a_{ii}X_{i}^{2} \end{array}$$

Where *Y* = predicted response, *a*_0_ = intercept coefficient, *a*_*i*_*X*_*i*_ = linear terms, *a*_*ij*_*X*_*i*_*X*_*j*_ = interaction terms and *a*_*ii*_*X*^2^ = square terms.

It has been shown that the RSM model is simple, efficient, less time consuming and capable of predicting the optimization of various processes of metabolite production. RSM is used to determine the factor levels which can simultaneously satisfy a set of desired specifications. This method helps us to determine, how a specific response is affected by changes in the level of the factors over the specified levels of interest and to achieve a quantitative understanding of the system behavior over the region tested. With the help of RSM we can predict the product properties throughout the region, even at factor combinations not actually run and to find conditions for the process stability. Combinations of PBD and RSM have been used in a number of studies for medium formulation to give optimum amount of desired metabolites. Singh and Tripathi, employed RSM for olivanic acid production and optimized the concentration of soybean meal, CaCO_3_ and glycerol and found 8-folds higher product formation as compared to the control un-optimized medium (Singh and Tripathi, 2008). By using minimum number of experiments and RSM methodology, 2-folds enhanced heparinase production was obtained by Banga & Tripathi, thus showed the importance of the method (Banga and Tripathi, 2009). Production of an anticancer drug actinomycin D from the submerged fermentation of *Streptomyces sindenensis* was found to be increased by 2.8-folds, when seven factor PBD was employed in the first step, followed by optimizing the concentration of the resultant efficient components through RSM in the second step (Praveen et al., 2008). These optimization techniques can also be employed in the improvement of performance of other microbial processes, like, biotransformation, fed batch fermentation, etc. Dubey et al. (2008, 2010) employed the statistical optimization to enhance the performance of biotransformation of colchicine into its pharmacologically active derivative 3-demethylated colchicine (3-DMC) through various microbial sources including recombinant *E. coli* under immobilized/non-immobilized condition (Dubey et al., 2008, 2010). They used CCD and RSM to optimize the three extraction variables (temperature, pH, and process time) and reported RSM as an efficient tool for the extraction of 3-DMC from the fermentation medium (Dubey et al., 2011). Similary, a group of researchers used RSM in fed batch fermentation condition to improve the production of jiean-peptide (JAA) from *Bacillus subtilis* and found 44% enhanced yield of JAA in comparison to the production under batch fermentation (Zhong et al., 2014). Likewise, Ghasemi et al. (2011) used RSM technique for improving the extraction performance of various components of essential oils, such as α-pinene (31.8%), 1,8-cineole (24.6%), limonene (14.8%), linalool (8.3%), and α-terpinolene (4.8%), present in the leaves of Myrtus communis. By using optimum conditions achieved through RSM technique, the percentage of the three components reached more than 85% of the crude extract.

Even though widely employed with much success, some limitations are associated with RSM, for e.g., the prediction of responses based on second-order polynomial equation is often limited to low levels and results in poor estimation of optimal formulations (Baishan et al., 2003). Another important limitation is the metabolic complexity of the microorganisms. When a large number of variables are involved, the development of rigorous models for a given biological reaction system on physical and chemical basis is still a critical challenge. This is probably due to the non-linear nature of the biochemical network interactions and in some cases the incomplete knowledge about the kinetics involved in such systems (Franco-Lara et al., 2006). Also, it is quite complicated to study the interactions of more than five variables and large variations in the factors can give misleading results possibly due to error, bias, or no reproducibility. To overcome the limitations of RSM another technique, nowadays ANN has been widely used by the researchers.

##### Artificial neural network

**Figure 3:**
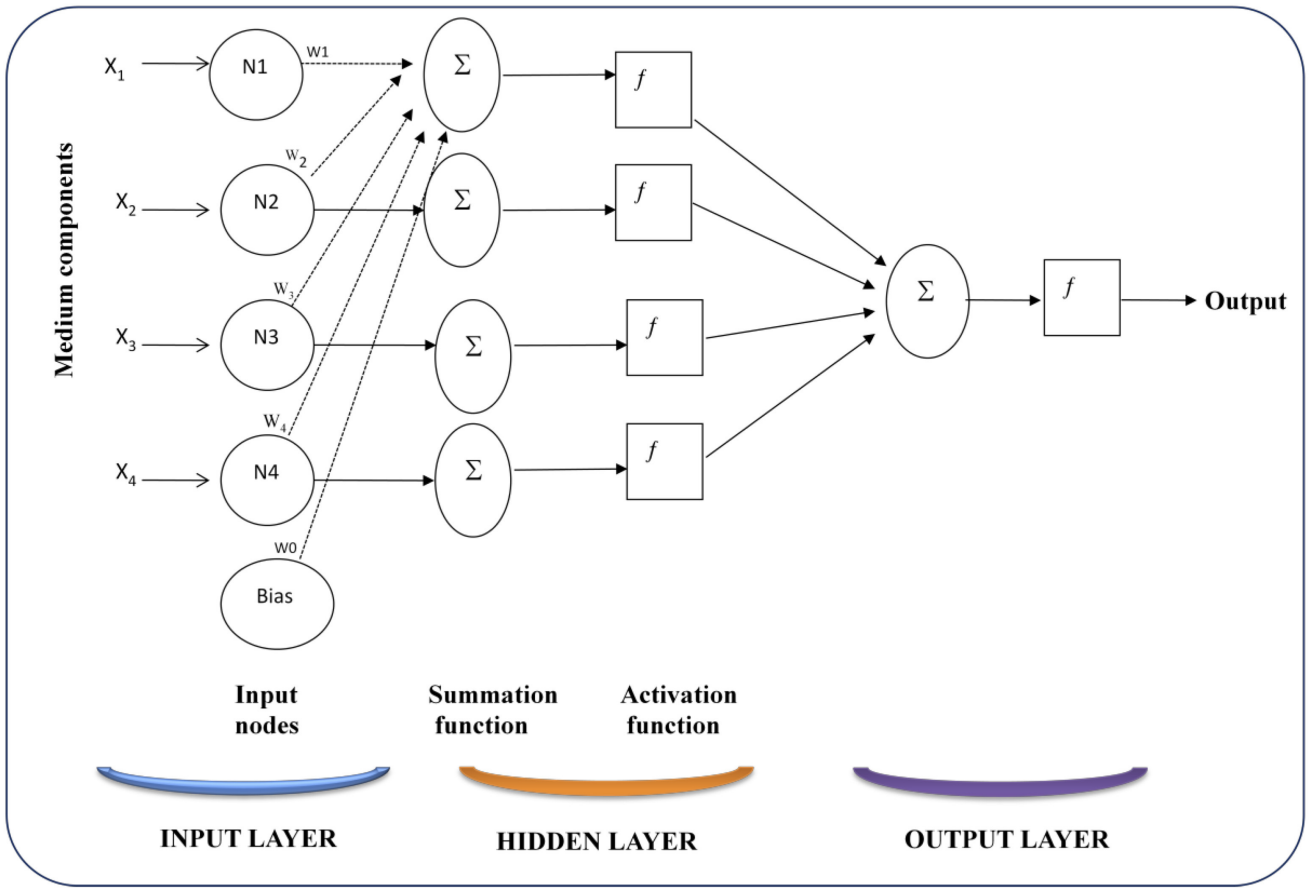
**Multilayer feed forward network with one hidden layer**.

An artificial neural network (ANN) is a mathematical or computational model that is influenced by the structural and/or functional aspects of the biological neural networks. Neural networks are typically applied in the estimation and multi-step prediction problems, but can also be used as controllers directly or as an adjuster of any process parameter for a conventional controller. ANN mimics the learning ability of the brain (Bhagat, 1990), and consists of input (like synapse), which are multiplied by weights (strength of respective signals) and then computed by a mathematical function which determines the activation of neuron. In most cases ANN represents an adaptive system that changes its structure according to external or internal information that flows through the network during the learning phase. They are simply “trained” using a data set and then applied to predict new data points. Prior knowledge or equations is not essential for this training as the network and system remains as a black box to the user. Significant characteristics of ANNs are that they can work smoothly with large amounts of data, excel at complex pattern recognition and require no mechanistic description of the system (McCord-Nelson and Illingworth, 1991). ANN is well suited for medium design, as it generates a large amount of data that often contains hidden pattern. The architecture of the ANN consists of three layers of information known as neurons: a layer of “input” units is connected to a layer of “hidden” units, which is further connected to a layer of “output” units (Figure 3). The “learning conditions” of neural networks are classified into three groups as *supervised* (*associative*), where the neural network is trained by giving it input and output experimental data. *Unsupervised* (*Self-organization*) in which output unit is trained to respond against clusters of pattern within the input. Different from the supervised, there is no prior set of groups into which the patterns are to be classified; rather the system must develop its own representation of the input stimuli. *Reinforcement* where learning may be considered as an intermediate form of the above two classes of learning. The learning system categorized its action as good or bad based on the environmental response and accordingly adjusts its parameters. Generally, the parameter adjustment is continued until the attainment of an equilibrium state.

ANNs have been widely applied with great success for system designing, modeling, optimization and control mainly due to its capacity to learn filter noisy signals and generalize information through a systematic training procedure (Foster and Katz, 1981; Singh et al., 2009). The optimization techniques are mostly general techniques and can be employed in various fermentation processes with similar efficiency. Osama et al. (2013) used ANN technique to optimize nutrient mist reactor for hairy root growth and developed an efficient model for optimizing the culture conditions and also predicted the biomass productivity effectively under different culture conditions. In another study, a combination of ANN and genetic algorithm (GA) was applied for maximizing the native concentration and shelf life of aspartate-β-semialdehyde dehydrogenase protein (Khan et al., 2011).

Neural network can perform on problems which have non-linear programs/relationships. When an element of the neural network fails, even then it can continue working without any problem by their parallel nature (Vaidya et al., 2003). It can be implemented in any application without any problem and doesn't need to be re-programmed. There are certain limitations of neural networks, for e.g., it needs proper training to operate efficiently. In ANN, the quality of the input data for training decides the quality of the output data.

##### Genetic algorithm (GA)

A trained mathematical model serves as a fitness function in the determination of optimum concentration of the medium components using GA. GA mimics the process of mutation and is based upon the principle “survival of the fittest”. This algorithm is based on the biological process of evolution, i.e., natural selection (Houck et al., 1995). The GA repeatedly modifies a population of individual solutions. At each step, the GA selects some of the individual solutions at random from the current population as parents. It then uses the selected ones to produce the off springs for the next generation, thus over a successive generations, the population “evolves” toward the most favorable solution. One can apply GA to solve a variety of optimization problems that are not well suited for standard optimization algorithms (Franco-Lara et al., 2006), including problems in which the objective function may be non-differentiable, discontinuous, stochastic or highly nonlinear. The GA follows mainly three types of rules at each step to create the next generation from the current population: *Selection rule* selects the individuals, known as parents that contribute to the population of the next generation. *Crossover rule* combines two parents to form children for the next generation. *Mutation rule* applies random changes to individual parents to form children. GA was successfully used to optimize medium composition for rifamycin B production using mutant strain of *Amycolatopsis mediterranei* at shake flask level (Bapat and Wangikar, 2004). By using ANN coupled GA method, Singh et al. (2009), designed an optimized the media for actinomycin V production by using a newly isolated strain of *Streptomyces triostinicus* and reported 4-folds (yield 452 mg/l) higher yield in optimized media in comparison to the normal production medium (yield 110 mg/l). One of the major advantages of GA is, it can handle a large amounts of data with no new guessing at each experiment, as the direction is automatically set. Hence, GA is the best method for solving complex optimization problems.

##### Nelder–Mead simplex

Nelder Mead (NM) simplex method is another statistical technique, which has been found to be helpful in reducing the expenses of classical optimizations and gives satisfactory results. NM simplex method is based on a real-parameter black-box optimization method and works well with irregular objective functions. The “term” simplex denotes a regular-sided figure in *n* + 1 dimension. For two dimensions, the simplex should be an equiangular triangle and for three dimensions, it should be a tetrahedron. NM simplex method for the function of *n* parameters compares the objective function at the *n* + 1 vertices of a simplex and gives the worst vertex through stepwise simplification search (Kennedy and Krouse, 1999). During this process, the direction of betterment is achieved by shifting the results away from the highest point with the smallest value. During optimization process to maximize lipid production, full factorial and multiple linear regressions were used to fit the polynomials to the data obtained (Kennedy and Krouse, 1999).

The NM simplex method frequently gives significant improvements in the primary iteration and produces quick and satisfactory results. This technique can also be successfully used in combination with ANN to optimize the production of various metabolites. Overall an improvement in the function value is more practical rather than full optimization (Singer and Nelder, 2009). Estimation of the process parameters and process controls are some of the practical problems, where the function values, are uncertain. Therefore, a high level of accuracy in solution is not necessary, and may be impossible to compute. The production of actinomycin D from *Streptomyces sindenensis* under submerged fermentation conditions is one of the best example, where the above combination has been used (Tripathi et al., 2012). They compared the results of ANN-GA combination with ANN-Nelder-Mead downhill simplex (NMDS) optimization and reported that later was more efficacious and gave roughly 12% higher yield than the yield obtained by ANN coupled with GA under the same conditions. Optimum shake-flask conditions were further optimized at bioreactor level (Khan and Tripathi, 2011). They used GA and NMDS separately to optimize the fermentation parameters, like, air flow rate and stirring rate of bioreactor for maximum actinomycin D production. Almost similar optimum combination of fermentation parameters were predicted by GA and NMDS. Nearly, 1.5-folds actinomycin D production was increased as compared to the optimum point in a shake-flask experiment (1.26 to ~2 gm/L).

## Problems and bottle necks in medium optimization techniques

Medium optimization involves large number of experiments irrespective of media chosen, which accounts for labor cost and is an open ended experiments. Rarely, the data generated from the shake flask media match exactly with the fermenter studies (Kennedy et al., 1994; O'Kennedy et al., 2003). All shake flask studies suffer from four main weaknesses, pH cannot be controlled, poor oxygen transfer capabilities, inadequate mixing and considerable evaporation during the process. It is widely assumed that the best medium obtained in the shake flask culture method will be the best media in the fermenter. Unfortunately, not many rigorous studies regarding the comparison of medium performances at different scales have been carried out in this line (Gupta and Rao, 2003). Furthermore, the industrial scale medium usually suffer from the problems such as batch-batch variability, availability all around the year, fluctuations in the price, stability during the transport time cost, problems associated with bulk storage and time.

Microbes or cells are dynamic in nature with lot of internal control mechanisms, but most media optimization studies treat them as black box or utilized solely for empirical data only. We believe that the next generation of medium optimization techniques should take the metabolic pathway regulatory mechanism into consideration. Not only that even the rate of mutations that occur in the particular medium under the influence of medium components should also be considered, as they might increase or decrease the yield or product which we are interested. If mutant strains are available they should also be explored in the medium optimization studies, as they might give us a way to develop new process, where a totally new cheap medium can be used.

The most important thing is, various optimization studies are focussed on the liquid culture based fermentation, but there are no such extensive methods available for solid or semi-solid state fermentation techniques. Almost all the researchers encounter this problem, “when should one stop applying the further optimizations techniques or which step is the end point of optimization studies” at one stage or other. Designing a fermentation medium can be a never ending problem, as the final endpoint, e.g., yield is an arbitrary value, which is depended upon various other factors. Most experts in the fields always look out for new components or media to increase the yield.

## Future directions in optimization techniques in designing of fermentation medium

In addition to the strain improvement strategies, medium optimization has been proved to be another valuable strategy toward the enhancement of product yield and process improvement. Evolution of medium formulations through screening of various carbon and nitrogen sources and their different combinations can significantly improve microbial growth, viability and overall yield of product during process development. Fermentation product cost could be reduced by replacing expensive components with cheaper sources and/or by increase in productivity. These are the goals of a successful optimization strategy. There are still some points which need to be considered for more precision and further optimizations, for e.g., every microbe has some limitations at their gene level for the production of specific metabolite, thus search for a new microbe with greater productivity is always required. Sometimes microbes in the present conditions are not able to utilize the cheaper raw material but through mutation it might be possible to make them able to assimilate low cost substrate with better performance. Genetic manipulation is the alternate way to increase the productivity of the microbes. Recent concerns about the genetically modified microorganism have put a big question mark on the use of recombinant microbes in large scale fermentation. Hence, the use of natural microbes is of great choice for various researchers and industrial personnel.

As substrate limitation condition is the key factor of secondary metabolite production therefore designing and optimization of chemostat mode of production may increase the productivity and reduce the loss of unused substrate. Further designing of mist or fluidized bed bioreactor is the alternate to reuse the microbe in long term and maximum utilization of substrate. It is difficult to understand the precise nature of the microbe or the other living system and the biology but with increase in understanding it will be feasible to select suitable design for better performance; for e.g., metabolic flux is the turnover rate of any product or molecules formed through a metabolic pathway which is regulated by a series of enzymes involved in that particular pathway. Detailed knowledge of metabolic flux and its regulation could be helpful to design a medium or the performance of mathematical models with greater accuracy. Moreover, prior knowledge of the biosynthesis of the desired metabolites provide the information about the intermediate(s) formed during the biosynthesis of that metabolite which in turn will be helpful during the selection of carbon, nitrogen or salt solution which can also act as an inducer for the production of desired metabolites.

## Conclusions

Optimization of the fermentation media is an essential step for metabolite production prior starting with semi-pilot/pilot production plans. In this critical review, conventional, and advanced optimization techniques used in medium optimization process have been reviewed and discussed. The statistical approaches were found to have potential to save experimental time for the process development and quality improvement. Also, optimization techniques help in reducing the overall product cost. The designs and methods discussed in this review have been analyzed on the basis of efficiency, simplicity and time consumption, and their applications have been suggested accordingly. However, the medium formulated after employing various designs still needs further evaluation under realistic production conditions and lastly with full scale models that reflect the production environment. Overall, this review provides a rationale for the selection of suitable updated technique for the media optimization employed during the fermentation process of metabolite production. Also, in recent years, a novel approach of integrated mode of microbe cultivation, i.e., aerobic and anaerobic fermentation using suitable facultative microbes have been tested and applied. Cheng et al. (2014) has applied this integrated approach for the production of xylitol and ethanol by using *Candida tropicalis*, and reported that under aerobic conditions xylitol is the end product that acts as a substrate for ethanol production by anaerobic cultivation. Likewise, Huang et al. (2014) suggested that switching of anaerobic/aerobic conditions during cultivation stage affect the bacterial community composition and subsequent degradation of chlorophenols in biocathode microbial fuel cells. This integrated approach has several advantages, for e.g., no supplementary operation unit needed, zero wastewater generation, processing on solid pre-treated material, and also, no need for sterilization; all of these advantages can help make the biotechnology industry more economical and environmentally friendly.

## Author contributions

Conceived and designed the study and experiments: VS, SH, RN, AS, MP, CT. Performed the experiments: VS, SH, RN, AS. Analyzed the data: VS, SH, RN, CT. Contributed reagents/materials/analysis tools: RN, AS, MP, CT. Wrote the paper: VS, SH, MP, CT. All authors reviewed the manuscript.

## Funding

VS is thankful to the Department of Science and Technology, Ministry of Science and Technology, Government of India, New Delhi, India for the fast-track fellowship (SR/FT/LS-190/2009).

### Conflict of interest statement

The authors declare that the research was conducted in the absence of any commercial or financial relationships that could be construed as a potential conflict of interest.
